# Functional Traits Explain Variation in Chaparral Shrub Sensitivity to Altered Water and Nutrient Availability

**DOI:** 10.3389/fpls.2019.00505

**Published:** 2019-04-18

**Authors:** Reina L. Nielsen, Jeremy J. James, Rebecca E. Drenovsky

**Affiliations:** ^1^ Biology Department, John Carroll University, University Heights, OH, United States; ^2^ Sierra Foothills Research and Extension Center, Browns Valley, CA, United States

**Keywords:** functional traits, nutrients, water, environmental change, drylands, chaparral

## Abstract

Worldwide drylands are threatened by changes in resource availability associated with global environmental change. Functional traits may help predict which species will be most responsive to these alterations in nutrient and water availability. Current functional trait work focuses on tissue construction and nutrient concentrations, but plant performance in low resource environments also may be strongly influenced by traits related to nutrient budgets and allocation. Our overall objective was to compare trait responses in a suite of serpentine and nonserpentine congener pairs from the California chaparral, a biodiverse region facing nutrient deposition and future changes in precipitation. In a common garden greenhouse environment, we grew small plants of *Arctostaphylos manzanita, A. viscida, Ceanothus cuneatus, C. jepsonii, Quercus berberidifolia,* and *Q. durata* in contrasting soil nutrient and moisture treatments. We measured a suite of traits representing physiological, growth, and mineral nutrient responses to these treatments. Overall, plant growth rate and leaf-level phosphorus use efficiency were greatest in the low water, high nutrient treatment, and lowest in the high water, low nutrient treatment. Variation in growth rate and plasticity among species and treatments was primarily associated with differences in mineral nutrition-based traits as opposed to differences in biomass allocation or specific leaf area. Namely, faster growing species and species with greater plasticity allocated more nitrogen and phosphorous to leaves and demonstrated greater photosynthetic phosphorus use efficiency. Overall, nonserpentine species had greater plasticity and biomass response to resource addition than serpentine species, and congener pairs responded to these resource additions more similarly to each other than species across congener pairs. This study extends our general understanding of how functional traits may influence species responses to environmental change and highlights the need to integrate mineral nutrition-based traits, including allocation of nutrient pools and nutrient use efficiency into this larger trait framework. Ultimately, this insight can help identify, in part, why coexisting species may vary in sensitivity to anthropogenic driven changes in soil resource availability.

## Introduction

Increased drought intensity due to rising temperatures is expected to have pronounced effects on drylands across the globe (International Panel on Climate Change, 2013; Prăvălie, 2016), with the areal extent of drylands projected to increase, ultimately covering nearly one-half of terrestrial systems worldwide by the end of the century (Huang et al., 2016). In many dryland systems, these anthropogenic-driven changes in water stress may be overlaid with substantial changes in soil nutrient availability due to a number of factors, including atmospheric deposition, altered disturbance regimes, and agricultural intensification (Fenn et al., 2003a; Millennium Ecosystem Assessment, 2005; Fenn et al., 2010; Huang et al., 2017). As drylands are dominated by slow-growing, resource-conserving plants, substantial changes in drought and nutrient supply have the potential to rapidly alter species abundance and community composition and ultimately ecosystem-level processes driven by plant community composition (Fenn et al., 2003b). While several long-term and short-term studies have documented pronounced changes in dryland plant community composition following nutrient enrichment or a combination of drought and nutrient enrichment, in most cases, these responses appear to be highly variable among species (e.g., Báez et al., 2007; Allen et al., 2010; Kinugasa et al., 2012; Vourlitis, 2017), and we largely lack a physiological foundation for predicting how these simultaneous anthropogenic stressors may impact co-occurring native species. Such a framework is ultimately necessary to link variation in climate change exposure to differences in species and plant community vulnerability to environmental change (Shi et al., 2018).

Plant functional traits are increasingly viewed as a robust framework to understand variation in species distribution and response to environmental change (Funk et al., 2017; Costa et al., 2018; Griffin-Nolan et al., 2018), and this framework may provide a strong platform to generate insight on how simultaneous changes in drought and nutrient availability may differentially impact co-occurring dryland species. To date, the bulk of the functional trait framework has focused on the economics of tissue construction and its impacts on stress tolerance, growth rate, tissue longevity, and plasticity (Wright et al., 2004; Chave et al., 2009; Onoda et al., 2011; Fridley et al., 2016). Although these traits may play important roles in determining competitive hierarchies among fast- and slow-growing species (Funk et al., 2017), they miss key traits related to plant nutrient budgets, storage, and conservation, which are critically important for plants from resource-limited systems. For example, plants from low resource environments allocate more biomass to storage tissues (i.e., stems, roots) relative to plants from high nutrient environments. As a result, changes in leaf-level traits may have less influence on whole plant nutrient conservation in low nutrient adapted species (Silla and Escudero, 2003). Therefore, focusing functional trait analyses on resource conservation traits, such as tight internal nutrient recycling, high nutrient use efficiency, greater root investment, and higher nutrient storage capacity, may be more important than simple leaf-level analyses for understanding plant responses to environmental change in low resource systems. This whole plant perspective is crucial, as multiple traits can influence mean nutrient residence time and can act in compensatory or synergistic ways. For example, by internally recycling nutrients through resorption, fewer nutrients need to be absorbed from the soil, but this process requires investment in nutrient mobilization and storage (Silla and Escudero, 2003). Based on these trade-offs, either root nutrient absorption or leaf nutrient resorption may be the best strategy for increasing mean nutrient retention time, depending on soil nutrient availability (Wright and Westoby, 2003). Thus, multiple traits influence mean nutrient residence time, but they may come at a cost if investment in one trait decreases depending on another—i.e., the traits may be compensatory.

Furthermore, given their typically low plasticity (Lambers and Poorter, 1992), low resource adapted plants may be unable to alter trait responses under environmental change scenarios to maintain fitness and survivorship. However, little research has probed how these species may respond to multiple environmental change factors. For example, greater root investment for acquiring soil resources should increase storage capacity. However, some work suggests trade-offs in the safety of plant water transport systems and the ability to store water and nutrients in stems and roots (Pratt et al., 2007). As a result, drought may increase the need to recycle other nutrient sources (e.g., through the process of leaf nutrient resorption). Therefore, if we are to understand the potential impacts of environmental change on low resource adapted plants, we must broaden the range of traits measured, include multiple resource manipulations in our studies, and explicitly assess intraspecific trait plasticity.

The California Floristic Province, a priority hotspot for global biodiversity conservation (Myers et al., 2000), is a particularly important system to understand how changes in drought and nutrient availability may differentially impact native species. Much of this province consists of drylands, where annual climatic water deficit and reoccurring drought strongly affect the distribution and abundance of species. Furthermore, mosaics of soil types varying in nutrient availability yield distinct plant communities with high species functional trait diversity. Among the most nutrient deficient soils in these mosaics are serpentine soils, derived from mafic and ultramafic rocks; these soils are rich in magnesium and heavy metals and deficient in critical macronutrients such as nitrogen, phosphorus, and calcium. In California, serpentine soils account for 1.5% of total land area but support 13% of the endemic species (Safford et al., 2005). In many cases, this high level of endemism includes closely related congeners species that are differentially distributed across these soil mosaics. Thus, these soil mosaics represent not only areas of key conservation concern but also provide an ideal system in which to ask questions of evolutionary adaptation and climate change sensitivity within a phylogenetic framework (Anacker, 2011). As a result, they may yield an improved understanding of how functional trait variation in nutrient-poor dryland systems influences how co-occurring species differ in their response to climate change exposure.

The overall objective of this work was to compare functional trait responses of related congeners originating from serpentine and nonserpentine chaparral communities of the northern California Coast Range in response to contrasting soil water and nutrient availability. These systems are predicted to experience changes in precipitation frequency and intensity (Cayan et al., 2008) and an overall increase in temperature as a result of climate change (Lenihan et al., 2003; Bachelet et al., 2016). Furthermore, urban and agricultural pollution threaten the California chaparral with atmospheric nutrient deposition (Fenn et al., 2003a, 2010; Bobbink et al., 2010). We sought to understand synergies and trade-offs among functional traits, as well as overall trait plasticity. We predicted that: (1) all species would respond positively to increased water and nutrients by increasing growth-related traits and having higher rates of gas exchange and nutrient use; (2) the relatively faster growing nonserpentine species would be more plastic in their trait expression than the very slow-growing serpentine species; and (3) that greater plasticity among the nonserpentine species would support greater trait compensation among nutrient conservation traits (e.g., greater investment in root biomass would decreases dependence on leaf nutrient resorption).

## Materials and Methods

### Site and Species Description

The University of California Donald and Sylvia McLaughlin Natural Reserve, Lower Lake, California, United States (38.82416, −122.335), is characterized by serpentine and nonserpentine soils that are dominated by chaparral shrublands, grasslands, and seeps. The climate is Mediterranean, with dry, hot summers and cool, wet winters with temperatures ranging from 40°C in the summer to below 0°C in the winter (University of California, Davis Natural Reserve System, 2003). Annual precipitation is ≈75 cm (30-year average; US Climate Data, 2017^1^). Large portions of the California Coast Range are adjacent to major urban areas or areas with intensive agriculture, resulting in pronounced local and regional anthropogenically caused atmospheric nutrient deposition (Fenn et al., 1998).

Three congeneric pairs of evergreen chaparral shrubs were used in this study. *Arctostaphylos manzanita* Parry, *Ceanothus cuneatus* (Hook) Nutt., and *Quercus berberidifolia* Liebm. grow on nonserpentine soil, whereas *A. viscida* Parry, *C. jepsonii* Greene, and *Q. durata* Jepson grow on serpentine soil. *Ceanothus* and *Arctostaphylos* species require specific cues for germination. *Ceanothus* species germinate in response to fire cues, whereas species of *Arctostaphylos* germinate in response to a combination of fire cues and scarification by acid in a mammalian gut. *Quercus* readily germinates and requires no special germination cues.

### Fruit Collection, Storage, and Seed Germination

Fruits of all species were collected from multiple populations at the McLaughlin Natural Reserve from at least 10 maternal plants per species. *Arctostaphylos* spp. and *Ceanothus* spp. fruits were collected in 2012 and stored at 28°C to promote after-ripening and maintain desiccated conditions, whereas *Quercus* spp. acorns were collected in winter 2016 and stored at 4°C to prevent germination prior to planting.

Because there are inherent differences in seed viability, germination requirements, germination percentages, and seed or fruit size, seed pre-treatments and germination conditions differed by species. Following protocols developed in our laboratory, *Arctostaphylos* spp. were germinated in fall of 2016, whereas *Ceanothus* spp. and *Quercus* spp. were germinated in January 2017. *Arctostaphylos* and *Ceanothus* seeds were removed from fruits by hand prior to treatments. *Arctostaphylos* spp. was scarified by soaking in concentrated sulfuric acid for 6 h. Seeds were rinsed in deionized water until pH paper indicated the rinse solution was neutral. Seeds were then soaked in <2% liquid smoke treatments for 12 h to imitate chemical cues found in wildfire smoke. *Ceanothus* spp. seeds were placed in boiling water for 6 min. To promote germination, seeds of *Arctostaphylos* spp. and *Ceanothus* spp. were germinated in nutrient agar under full spectrum growth lights (PPFD: 100 μmol m^−2^ s^−1^) in a laboratory at John Carroll University, University Heights, Ohio, United States. Upon first appearance of the cotyledons, seedlings were transferred into 4 × 14 cm deep seedling tubes (SC7R Ray Leach Cone-tainer, Stuewe & Sons, Inc.) with a mix of 1:1 sand and fritted clay mixture that contained 1 g of water storing crystals (Miracle-Gro Lawn Products, Inc) homogenized throughout the growing medium. *Quercus* spp. were planted immediately into 7 × 25 cm deep tree tubes (D40H Deepot, Stuewe & Sons, Inc.) containing sand and fritted clay and placed under full-spectrum Na halide growth lights (PPFD: 350 μmol m^−2^ s^−1^) in the greenhouse. When the specimens of *Arctostaphylos* and *Ceanothus* had at least three sets of true leaves and were able to withstand a higher PPFD without risk of rapid desiccation (≈3 months of growth), these species were moved to the greenhouse.

Within 1 week of planting in soil, all species were watered with a 10% modified Hoagland’s solution (Epstein, 1972) and a 10% Bonide Captan Fungicide solution to minimize fungal growth. A modified Hoagland’s solution was supplied twice more within the first month of growth. Fungicide was reapplied twice more after approximately one and two months of growth. Once the plants were well-established (April 13, 2017), all species were transplanted into deep, 2.83 L pots (TP414 Tall One Treepot, Stuewe & Sons, Inc.) to ensure sufficient rooting space for the duration of the study, and plants were allowed to adjust to these pots for 2 weeks prior to initial treatment.

### Experimental Design

Treatment initiation began on May 8, 2017. Species were assigned to treatments using a randomized complete block design with each species having 10 replicates for each of four treatments that were represented once per block. Treatments were: (1) high nutrients (N, P, K), high water; (2) low nutrients, high water; (3) high nutrients, low water; and (4) low nutrients, low water. High nutrient treatments consisted of 2 g of 10-10-10 slow release NPK fertilizer (0.2 g total N and 0.2 g P_2_O_5_, Miracle-Gro Lawn Products, Inc), whereas low nutrient treatments did not have any added fertilizer. Slow release fertilizer was applied once to the high nutrient treatments at treatment initiation to represent a natural spring nutrient pulse. High water represented a soil water capacity ≥18%, and low water treatments maintained a soil water capacity of ≈9%. These water treatments were chosen to mimic a very wet season that could be observed under climate change scenarios and a typical dry season and represent conditions observed in northern and southern California chaparral systems, including sandy and sandy loam soils, following soil recharge and during the dry season (Salve and Allen-Diaz, 2001; Jacobsen et al., 2009). Soil water availability was monitored three times a week using a Campbell Scientific Hydrosense II probe (Campbell Scientific Inc., North Logan, Utah). Initially for high water treatments, 250 mL of water was added if soil moisture was <18%, (see Khasanova et al., 2013). For low water treatments, no water was added if soil moisture was >9%, but 100 mL of water was added if the soil moisture was <9%. After 3 weeks of treatment, plant water demand had increased as a consequence of plant growth and longer day lengths; therefore, high water plants received 500 mL every day unless soil moisture capacity was >18%. This watering regime was followed until October 23, 2017, at which point all plants were allowed to slowly dry-down, mimicking end-of-season field conditions encouraging leaf senescence. Seasonal dry-down was encouraged by decreasing water addition treatments and maintaining the soil water capacity at lower levels. The high water treatment was maintained at 11%, and plants were given 100 mL of water when soil moisture levels were not met. The low water treatment was maintained at 9% soil moisture capacity, and plants were given 50 mL of water when soil moisture levels were not maintained. Some plants in the low water treatment were large enough to need 100 mL of water in order to maintain a 9% soil moisture capacity (Table 1). Also at this time, the greenhouse was set to cooler conditions, similar to winter months at the field site (daytime: 13–18°C; night: down to 7°C).

**Table 1:** Average soil water content (%) measurements of the two watering regimes during three phases of the experiment.

Experimental phase	High water	Low water
Spring initiation	10.30 ± 1.53	7.55 ± 1.04
Summer water increase	14.05 ± 1.82	7.97 ± 0.89
Fall drawdown	12.78 ± 1.08	8.46 ± 0.81

During the duration of this experiment, fungal growth and powdery mildew were evident on some plants. To combat fungal diseases, 100 mL of Bonide Captan Fungicide was applied four times throughout the experiment. When fungicide was applied, the 100 mL of water normally allocated for each daily treatment was substituted with the 100 mL of fungicide. If a sample did not need watering that day, fungicide was applied the next time water was required. When powdery mildew was observed, the affected leaf was treated with soapy water or 1% Bonide Rose Rx 3 in 1 solution, alternating when one was no longer effective, and then rinsed with small amounts of water.

### Pre-harvest Measurements

Physiological and morphological measurements were made on a subset of plants from each treatment. Gas exchange was measured on the youngest fully mature leaf using a LI-COR 6400XT Portable Photosynthesis System (LI-COR Inc., Lincoln, Nebraska, USA). Following measurement, the leaf was harvested and used to measure projected leaf area *via* image analysis (WinRhizo, Regent Instruments Inc., Saint-Foy, Quebec, Canada) in order to correct area-based gas exchange rates. Gas exchange measurements were measured midday on July 17, 2017 (24°C; sunny) and again on August 25, 2017 (22°C; sunny) when the plants were experiencing maximal seasonal growth. CO_2_ flow was set to 400 μmol s^−1^, the CO_2_ mixer to 400 μmol mol^−1^, and the light level in the LED chamber to a PPFD slightly above ambient conditions (1,300 μmol m^−2^ s^−1^). Three subsamples were taken at 10-s intervals for each replicate plant. IRGAs were matched every three plants. All leaves harvested at this time were dried and weighed to be included in further biomass analyses.

Stem height was measured four times throughout the experiment. Initial stem height was measured during treatment initiation. Stem height was measured from soil level to apical meristem. As soil levels shifted throughout the experiment, nail polish was used to mark the soil level on plant stems at the initial stem height reading. Stem height was also recorded on 8 July, 11 September, and 1 December, 2017, and was measured from the nail polish marking to the apical meristem. These measurements were used to determine relative growth rates (increases in stem height over time; mm day^−1^; Hunt, 1982) of each sample. Senescent leaves were collected throughout the fall drawdown treatment phase, and the date of collection was recorded.

### Harvest

A destructive harvest was performed on 1 and 2 December, 2017, at which point remaining senescent leaves were collected to determine nutrient resorption proficiency (*sensu* Killingbeck, 1996); these leaves were set aside for later analysis. Belowground biomass was separated from aboveground biomass at the soil level. Stem and leaf tissue were separated, and leaves were placed on ice in a cooler. Leaves were scanned for leaf area, as previously described. Soil was removed from belowground biomass, and all biomass was dried at 65°C until constant mass was achieved. Once dried, roots, stems, and leaves were briefly rinsed to remove excess soil or dust, allowed to dry for another 48 h at 65°C, and weighed separately. Leaves previously harvested for physiological measurements were included in the final leaf masses at this time, and specific leaf area (leaf area/leaf mass, SLA) was calculated. All biomass was ground using a Wiley mill, using a #40 mesh screen. Samples too small to be ground using a Wiley Mill were hand ground using a stainless steel mortar and pestle. Total stem, root, and green and senesced leaf nitrogen concentrations were analyzed *via* a CN analyzer (ECS 4010; Costech Analytical, Valencia, California, USA). Total green leaf, stem, and root tissue phosphorus were analyzed via ICP-OES (Plasma 400; Perkin-Elmer, Waltham, Massachusetts, USA), following dry-ashing and acid dissolution. Senesced leaf P was not determined due to low senescent leaf sample sizes. Senesced leaf N represents N resorption proficiency (*sensu* Killingbeck, 1996). Nutrient pools were calculated for green leaves, stems, and roots by multiplying organ biomass by tissue nutrient concentration.

### Growth, Gas Exchange, and Conservation Trait Calculations

Plant relative growth rate (RGR) was calculated as (height_final_/height_initial_/days since initial stem height measurement). Gas exchange and leaf nutrient data were used to calculate intrinsic water use efficiency (ratio of the amount of carbon gained per water lost; A/g_s_, μmol mol^−1^), photosynthetic phosphorus (P) use efficiency (ratio of carbon gain to leaf phosphorus; μmol CO_2_ mol P^−1^ s^−1^), and photosynthetic nitrogen (N) use efficiency (ratio of carbon gain to leaf nitrogen; μmol CO_2_ mol N^−1^ s^−1^). Phenotypic plasticity indices were calculated as ((maximum mean response-minimum mean response)/maximum mean response) for RGR, total biomass, specific leaf area, root mass ratio, instantaneous photosynthetic rate, photosynthetic N use efficiency, photosynthetic P use efficiency, intrinsic water use efficiency, senesced leaf N, total N pools, and total P pools (PI_v_ as described in Valladares et al., 2006). This index varies from zero to one, with low values representing traits showing little plasticity and high values representing traits with high plasticity.

### Statistical Analysis

To identify factors influencing physiological and morphological response variables, mixed-model MANOVAs were used. All treatment factors were included as fixed effects, and phylogenetic effects were included as random factors to account for potential evolutionary constraints on trait responses. MANOVA models included the following factors: congener pair (random effect), species (random effect), block (random effect), water treatment (fixed effect), nutrient treatment (fixed effect), and origin (serpentine or non-serpentine; fixed effect) as the main effects (see Funk et al., 2015). Interaction effects included the two-way interactions of water and nutrients, nutrients and origin, and water and origin, and a three-way interaction of water, nutrients, and origin. Physiological traits (instantaneous photosynthetic rate, intrinsic water use efficiency, photosynthetic N use efficiency, and photosynthetic P use efficiency), biomass related traits (RGR, total biomass, root mass ratio, and specific leaf area), and tissue chemistry (green leaf N and P) were grouped together for separate MANOVA models. Instantaneous physiological traits were averaged between the two dates of measurements. Assumptions of MANOVA (normal distribution and equal variance) were tested with a Shapiro-Wilks test and a Bartlett’s test, respectively. For the physiological MANOVA, data log10 transformed to better meet model assumptions. For each MANOVA model, *F* statistics, *P*-values, and Pillai’s trace are reported. Larger values for Pillai’s trace indicate a factor that contributes more strongly to the model. For some traits, low and unequal replication among trait responses prevented analysis by MANOVA, and individual ANOVA models were run for these traits, including leaf life span, senesced leaf N, total N pool, total P pools, and the proportion of N and P allocated to roots, using the same model structure for main and interactive effects as the MANOVAs. Total N and P pools were log10 transformed to better meet model assumptions. To compare coordinated trait responses, a principal components analysis (PCA) was run, including the following variables: RGR, root mass ratio, specific leaf area, photosynthetic N use efficiency, photosynthetic P use efficiency, intrinsic water use efficiency, total N pools, and total P pools. All analyses were run using the R statistical program (R coding team, 2016) using version 3.3.1 (2016-06-21), except the PCA, which was run using CANOCO v5 (Microcomputer Power, Ithaca, NY).

## Results

### Physiological Measurements

**Figure 1:**
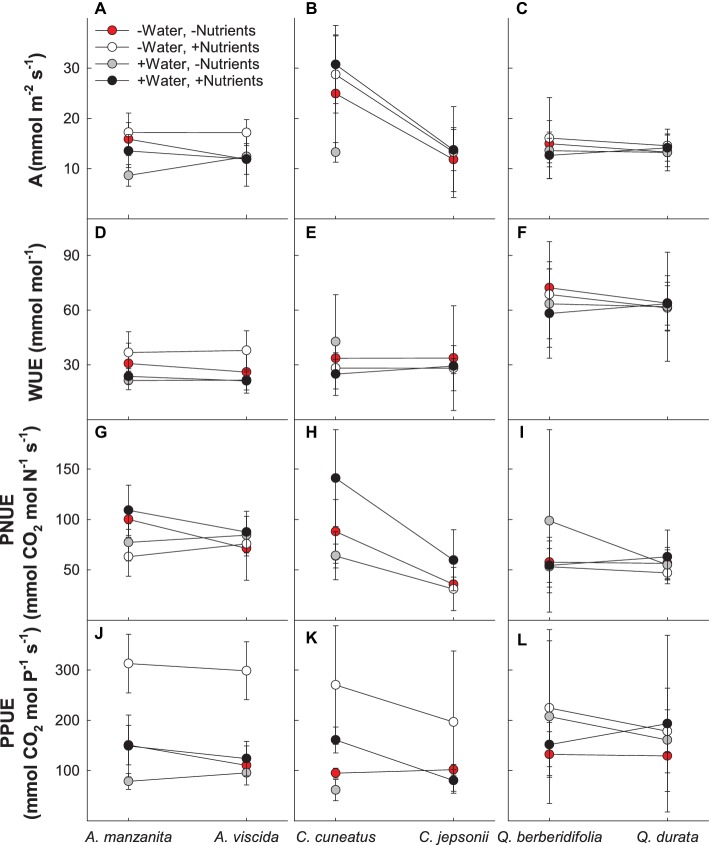
Gas exchange responses of plants exposed to contrasting water and nutrient treatments, including photosynthetic rate (A; **A–C**), intrinsic water use efficiency (WUE; **D–F**), photosynthetic N use efficiency (PNUE; **G–I**), and photosynthetic P use efficiency (PPUE; **J–L**). First plant in each congener pair originates from non-serpentine soil; second plant in each congener pair originates from serpentine soil. Data are means ± S.D. (*n* = 1–7).

Based on MANOVA results (Supplemental Table 1A), physiological responses depended on the combination of water and nutrient treatment the species received (Pillai’s trace = 0.239; *F_4,78_* = 6.137; *p* < 0.0001) and whether the species originated from serpentine or nonserpentine soil (Pillai’s trace = 0.135; *F_4,78_* = 3.041; *p* = 0.021). In general, the low water, high nutrient treatment plants tended to have higher photosynthetic rate and photosynthetic P use efficiency and lower photosynthetic N use efficiency than other treatment combinations, whereas the high water, low nutrient plants had low photosynthetic rate, photosynthetic P use efficiency, and intrinsic water use efficiency and moderate photosynthetic N use efficiency (Figure 1). Although not true for all traits, gas exchange–related traits tended to be lower in species originating from serpentine soil, particularly photosynthetic N and P use efficiencies (Figures 1G–L).

### Growth and Biomass Allocation

**Figure 2:**
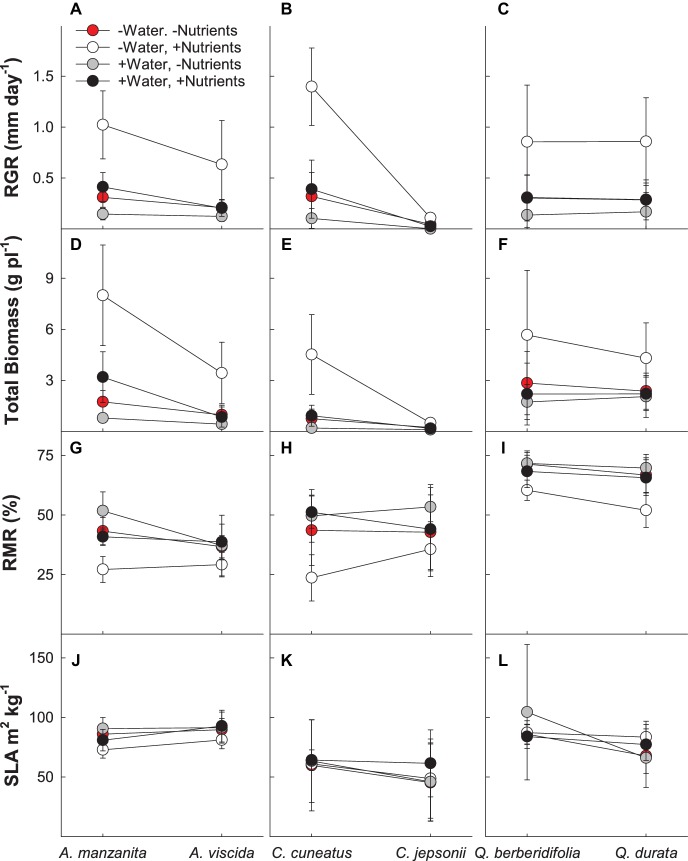
Growth and allocation responses of plants exposed to contrasting water and nutrient treatments, including relative growth rate (RGR; **A–C**), total biomass per plant **(D–F)**, root mass ratio (RMR; **G–I**), and specific leaf area (SLA; **J–L**). First plant in each congener pair originates from non-serpentine soil; second plant in each congener pair originates from serpentine soil. Data are means ± S.D. (*n* = 7–10).

In the MANOVA model, there were significant interactions between water and nutrients (Pillai’s trace = 0.177; *F_4,183_* = 9.86; *p* < 0.0001), water and origin (Pillai’s trace = 0.062; *F_4,183_* = 3.05; *p* = 0.018), and nutrients and origin (Pillai’s trace = 0.128; *F_4,183_* = 6.73; *p* < 0.001) for growth and biomass allocation measures (RGR, total biomass, root mass ratio, and specific leaf area; Supplemental Table 1B). In general, plants receiving the low water, high nutrient treatment had faster growth rates and a larger overall biomass (Figures 2A–F) than plants receiving all other treatments. Plants treated with high water and no added nutrients had the slowest RGR and lowest total biomass. Root mass ratio was lowest under the low water, high nutrient treatments and generally was highest in the high water, low nutrient treatment (Figures 2G–I). Nonserpentine species under low water treatments had the fastest growth rate and largest total biomass. When given additional water, both serpentine and nonserpentine species decreased in RGR, total biomass, and increased slightly in belowground allocation (Figures 2A–I). However, the serpentine species always had a slightly lower RGR and total biomass when given water than the nonserpentine species (Figures 2A–F). When only observing the nutrient treatments, nonserpentine species had a higher RGR and larger total biomass in the high nutrient treatment as compared to the low nutrient treatments of all species (Figures 2A–F). Specific leaf area was the least variable of the growth and biomass allocation measures (Figures 2J–L).

### Tissue Chemistry and Nutrient Pools

**Figure 3:**
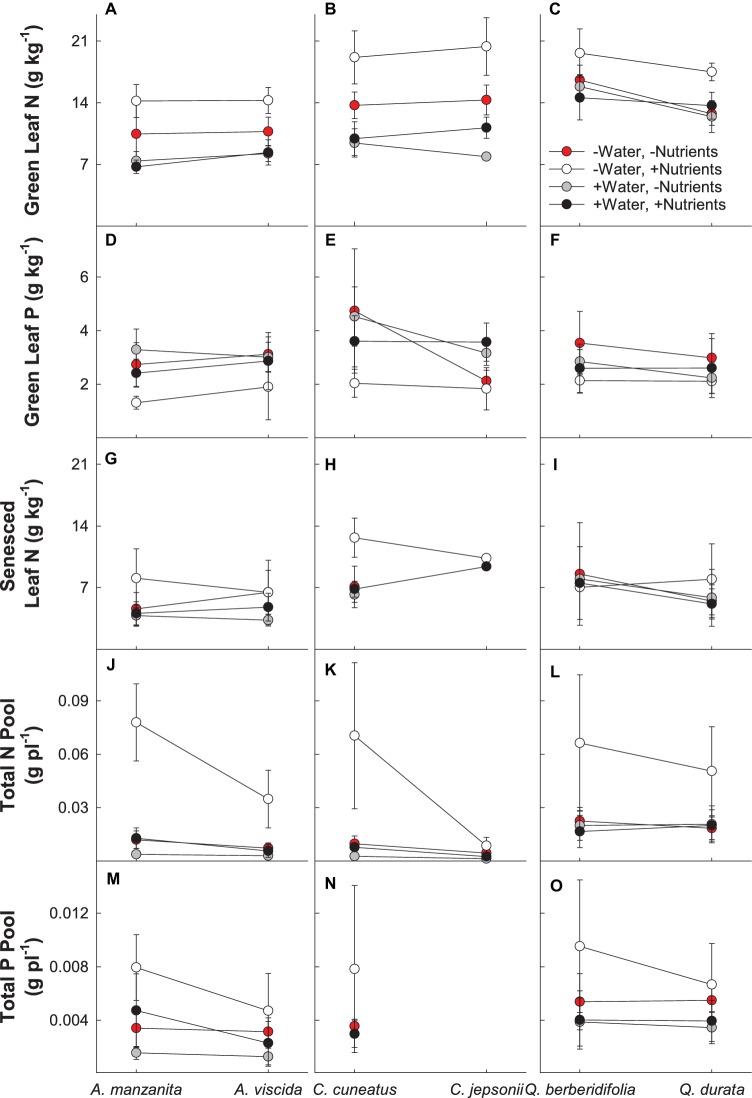
Plant nutritional responses to contrasting water and nutrient treatments, including green leaf N **(A–C)**, green leaf P **(D–F)**, senesced leaf N **(G–I)**, total N pool **(J–L)**, and total P pool **(M–O)**. First plant in each congener pair originates from non-serpentine soil; second plant in each congener pair originates from serpentine soil. Data are means ± S.D. (*n* = 1–10).

There were significant interactions between water and nutrients (Pillai’s trace = 0.237; *F_2,156_* = 24.208; *p* < 0.0001) and nutrients and origin (Pillai’s trace = 0.076; *F_2,156_* = 6.394; *p* = 0.002) for green leaf N and P in the MANOVA model (Supplemental Table 1C). For all species, the low water, high nutrient treatment resulted in the highest green leaf N, whereas the lowest concentration of N was observed in the plants under the high water, low nutrient treatment, as well as the high water, high nutrient treatments (Figures 3A–C). Green leaf P concentration was the lowest in the low water, high nutrient treatment (Figures 3D–F). Across all congeners pairs, green leaf N tended to increase and green leaf P tended to decrease with nutrient addition. However, the difference in magnitude of these changes depended on soil origin, with the magnitude of increase for green leaf N greater for the serpentine species and the magnitude of decline for green leaf P greater for the nonserpentine species (Figures 3A–F).

There was a significant two-way interaction between water and nutrients (*F_1,79_* = 4.24; *p* = 0.043) for senesced leaf N (Supplemental Table 2A). Generally, the low water, high nutrient treatments had the least amount of N resorbed (highest senesced leaf N), whereas the high water, low nutrient treatments resorbed the most N from their leaves (lowest senesced leaf N; Figures 3G–I). In general, plants receiving high nutrients began senescing leaves sooner than plants in the low nutrient treatments, with high nutrient plants beginning leaf senescence up to 23 days earlier than plants in other treatments (*F_1,115_* = 6.95; *p* = 0.0096; Supplemental Table 2B; data not shown).

**Figure 4:**
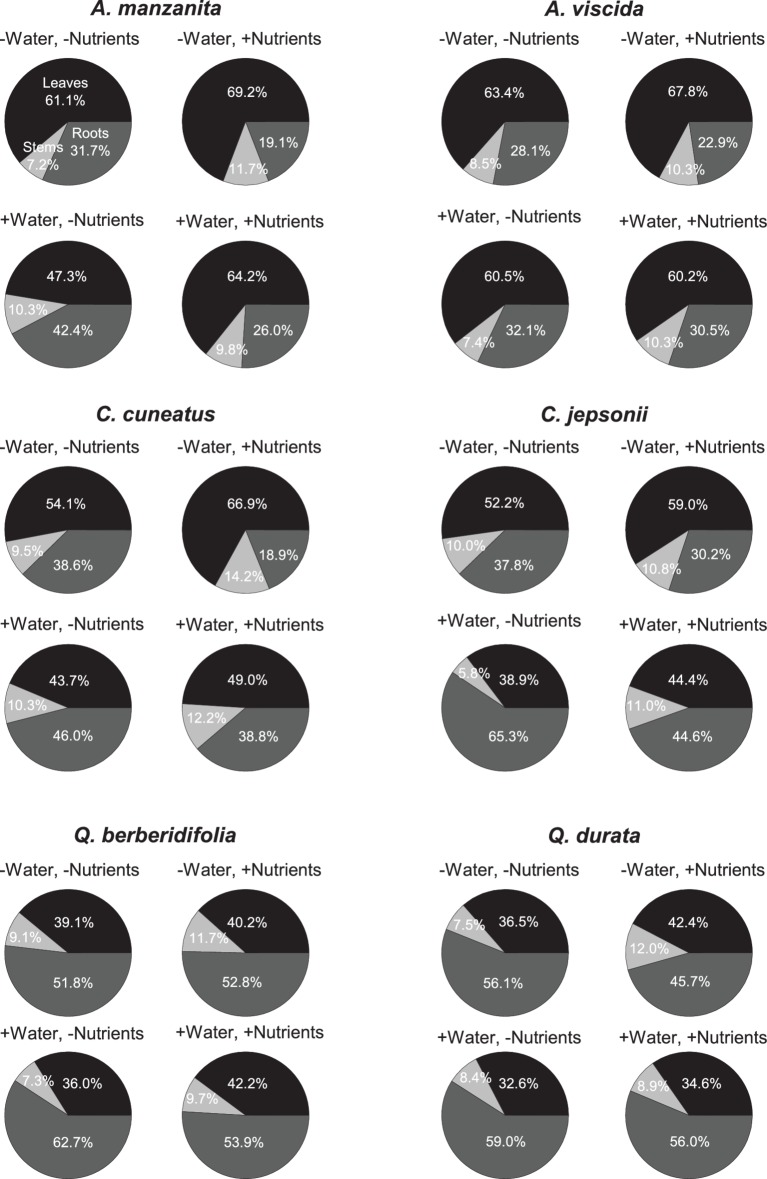
Proportional allocation of N to green leaf (black), stem (light gray), and root (dark gray) N pools. Taxa on left side of figure panel originate from non-serpentine soils, and taxa from the right side of the panel originate from serpentine soils. Data represent mean responses (*n* = 1–10).

**Figure 5:**
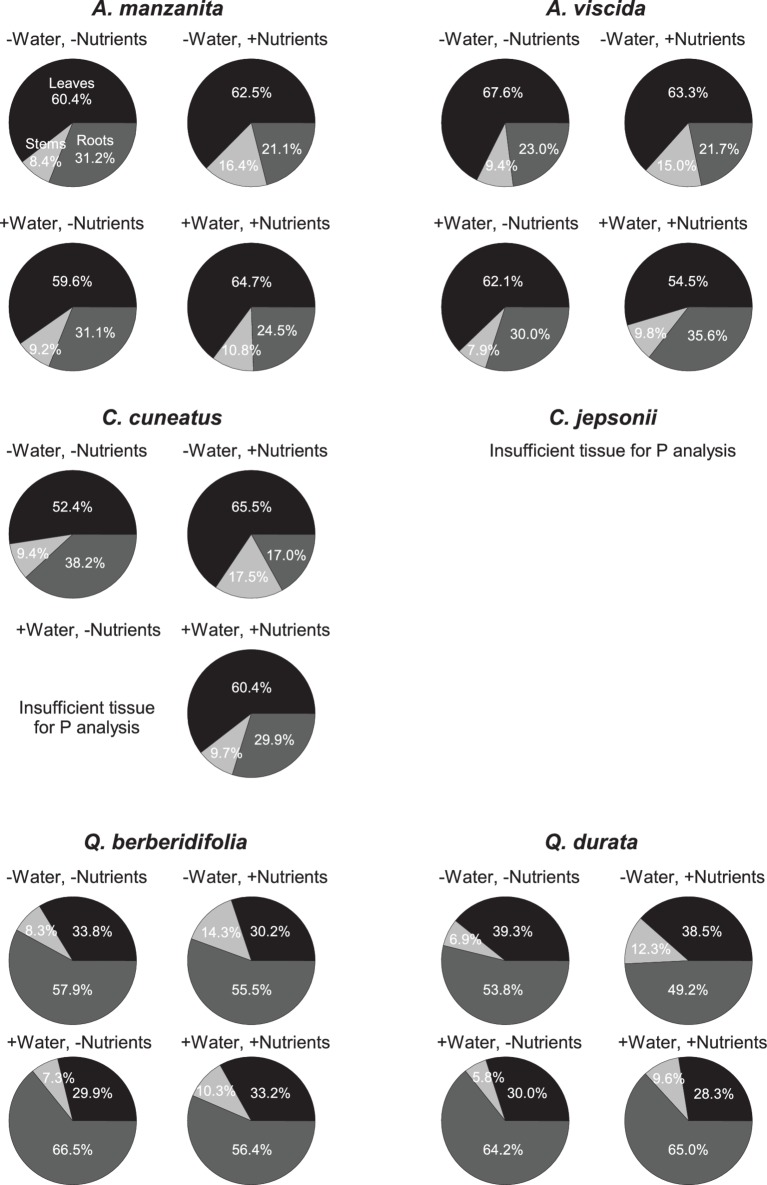
Proportional allocation of P to green leaf (black), stem (light gray), and root (dark gray) P pools. Taxa on left side of figure panel originate from non-serpentine soils, and taxa from the right side of the panel originate from serpentine soils. Data represent mean responses (*n* = 1–10).

Total N pools varied based on an interaction of water and nutrients (*F_1, 155_* = 22.29, *p* < 0.0001), nutrients and origin (*F_1, 155_* = 7.49, *p* = 0.007), and water and origin (*F_1, 155_* = 7.29, *p* = 0.008; Supplemental Table 2C). Total N pools tended to be smaller in species originating from serpentine soils, but across all species tended to be highest in the low water, high nutrient treatment (Figures 3J–L). Total P pools differed based on soil water availability (*F_1, 106_* = 36.21, *p* < 0.0001) and an interaction of nutrient availability and origin (*F_1, 106_* = 4.43, *p* = 0.04; Supplemental Table 2D). Total P pools tended to be larger in droughted plants (Figures 3M–O). Furthermore, nutrient addition tended to increase P pools, although the magnitude of this effect was greater in plants that originated from non-serpentine environments. Allocation of N to root pools was influenced by the water (*F_1,155_* = 27.61, *p* < 0.0001) and nutrient (*F_1,155_* = 32.14, *p* < 0.0001) treatments (Supplemental Table 2E). In general, allocation of N to root pools was greater in the high water treatments than the low water treatments and higher in the low nutrient treatments than high nutrient treatments (Figure 4). Allocation of P to root pools was influenced by significant interactions of water and origin (*F_1,106_* = 7.56, *p* < 0.007) and nutrient and origin (*F_1,106_* = 5.00, *p* < 0.03; Supplemental Table 2F). With water addition, serpentine species tended to increase allocation of P to roots more than non-serpentine species, and with nutrient addition, serpentine species tended to decrease allocation of P to roots more than non-serpentine species (Figure 5).

### Plasticity

**Figure 6:**
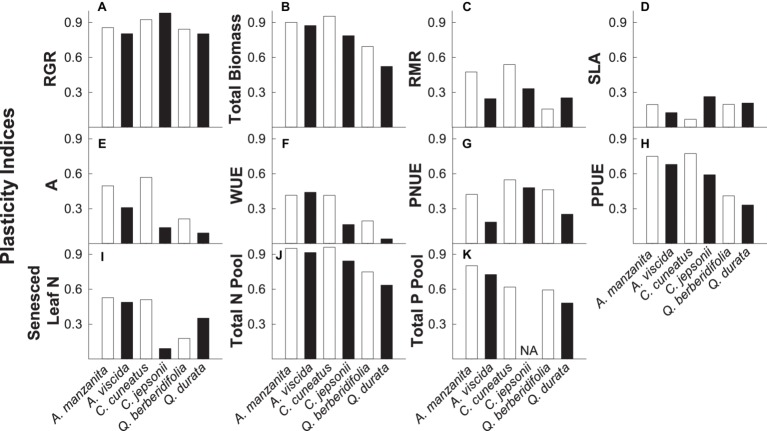
Plasticity indices (PI_v_) for relative growth rate (RGR; **A**), total biomass **(B)**, root mass ratio (RMR; **C**), specific leaf area (SLA; **D**) photosynthetic rate (A; **E**), intrinsic water use efficiency (WUE; **F**), photosynthetic N use efficiency (PNUE; **G**), photosynthetic P use efficiency (PPUE; **H**), senesced leaf N **(I)**, total N pool **(J)**, and total P pool **(K)**. A value of 1 indicates that the trait is completely plastic. White bars represent non-serpentine species, and black bars represent serpentine species.

Overall, nonserpentine species were generally more plastic than the serpentine species, and traits associated with growth rate, biomass accumulation, and nutrient pools (particularly total N pools) were more plastic than those associated with instantaneous physiological measures or green and senesced leaf N (Figures 6A–K). Additionally, qualitative inspection of PIv values suggested there was some evidence that congener pairs were similarly plastic for some traits, particularly RGR, photosynthetic P use efficiency, and total nutrient pools (Figures 6A,H,J,K).

### Coordination Among Trait Responses

**Figure 7:**
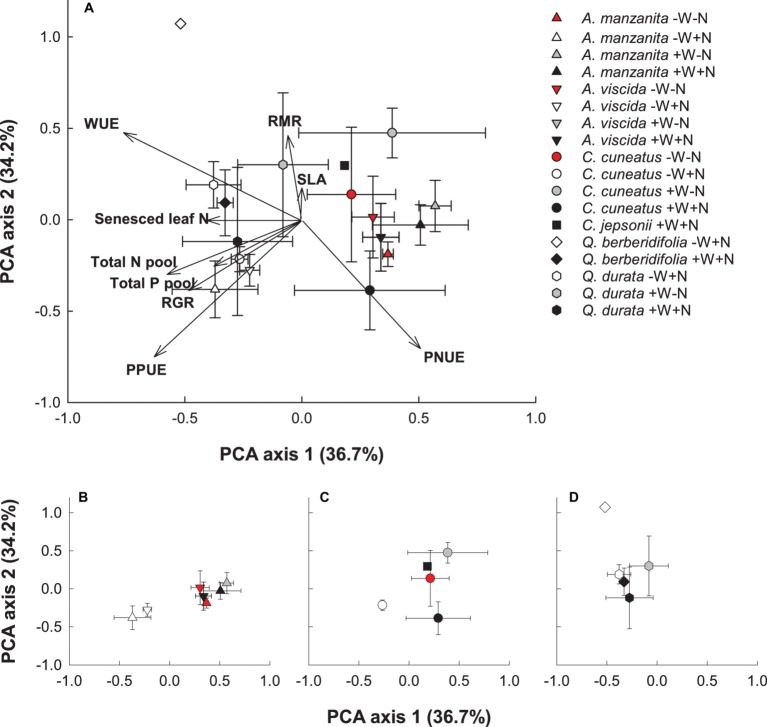
Principal components analysis (PCA) visualization of functional trait responses of focal taxa **(A)**. Centroids are species means ± S.D. (*n* = 1–4). Congener responses presented separately in smaller panels: *Arctostaphylos* **(B)**, *Ceanothus* **(C)**, and *Quercus* **(D)**. *A. manzanita* (up triangle), *A. viscida* (down triangle), *C. cuneatus* (circle), *C. jepsonii* (square), *Q. berberidifolia* (diamond), and *Q. durata* (hexagon). Low water, low nutrients (red); low water, high nutrients (white); high water, low nutrients (gray); high water, high nutrients (black). Due to missing trait values for some replicates, not all species by treatment combinations were retained in the analysis.

Based on the PCA visualization of trait responses, species responded more strongly to water and nutrient addition than phylogenetic influences (Figures 7A–D). Plants experiencing the low water, high nutrient treatments plotted to the left of the first axis and were most strongly associated with higher senesced leaf N (i.e., poorer resorption proficiency), RGR, total N and P pools, intrinsic water use efficiency, and photosynthetic P use efficiency. Plants from the other treatments showed less consistent trait responses, with the *Quercus* species tending to group close together regardless of treatment (Figure 7D). The first axis was most strongly associated with senesced leaf N, a measure of nutrient resorption proficiency, and the second axis was most strongly associated with specific leaf area and root mass ratio.

## Discussion

### Functional Trait Responses to Water and Nutrient Addition

Contrary to our initial hypothesis, all species did not respond most positively to increased water and nutrients. Instead, plant performance was often greatest in the low water, high nutrient treatment and lowest in the high water only treatment. Plants in the low water, high nutrient treatment tended to invest less biomass belowground and achieved the highest RGR and total biomass, contrary to what we might expect under droughted conditions (Chapin, 1980). These plants also had the largest nutrient pools, and they invested a greater proportion of their nutrient pools aboveground. Allocation aboveground, rather than into roots, promotes future carbon gain and thus a greater return on investment (Drenovsky and James, 2010), which likely supports higher biomass accumulation in this treatment. If our pot conditions are a reasonable simulation of nutrient and water availability in the field, our data suggest that these chaparral species could respond positively to future nutrient deposition, but these positive responses could be dampened during periods of high precipitation. In contrast, high water treatments had negative impacts on plant performance, particularly under low nutrient conditions and for the serpentine species, suggesting that increased precipitation may be a greater threat to plant success under future environmental change scenarios than nutrient deposition. However, further work is needed, particularly under field conditions, testing how these responses might change with ontogeny and root system size or depth.

Compared to other treatments, plants had higher green leaf nitrogen and lower green leaf phosphorus in the low water, high nutrient treatment. As a result, plants experiencing this treatment had a high photosynthetic P use efficiency, an important trait especially for species originating from serpentine soils, which tend to be very low in P where the seeds for the species were collected (Drenovsky et al., 2013). These differences in leaf chemistry could be due to the mobility of these nutrients. Nitrogen, which has a high mobility, can easily be taken up from the soil, as transpiration in these species was maintained even under droughted conditions (data not shown). In contrast, phosphorus is relatively immobile, thus limiting the amount of phosphorus that could be absorbed, especially in low water conditions (Marschner, 2012). It is also possible that the low green leaf phosphorus observed in the low water, high nutrient treatment could be the result of biomass dilution (Jarrell and Beverly, 1981), as these plants also had the largest overall biomass.

Not only did the treatments affect green leaf tissue chemistry but also resorption proficiencies of these species. The species given the low nutrient treatments, particularly the high water, low nutrient treatment tended to be very proficient resorbers, often exhibiting complete resorption (Killingbeck, 1996). However, the species in high nutrient treatments tended to resorb less, implying that these species rely less on resorption as a nutrient conservation strategy when soil nutrients are high. Because resorption is an energy intensive process (Chapin and Kedrowski, 1983), plants exhibiting intermediate or incomplete resorption will have more energy available to spend on other processes, such as growth, as was observed in all our species.

### Plasticity Responses to Water and Nutrient Additions

In partial support of our hypothesis, the nonserpentine species, overall, showed greater functional trait plasticity, with differences in congener pairs being most substantial between the *Arctostaphylos* and *Ceanothus* species, while differences between the *Quercus* species were less distinct. Higher overall plasticity in faster-growing species is to be expected, as plants from higher resource areas are better adapted to take up nutrients when they become available (Funk, 2008), unlike slow-growing plants, which tend to have a steady rate of uptake regardless of nutrient availability (Chapin, 1980). Species were the most plastic in traits related to growth and nutrient pools, moderately plastic in traits related to gas exchange and resorption proficiency, and least plastic in traits related to morphology and allocation (e.g., root mass ratio and specific leaf area). Gas exchange data were highly variable within species and treatment, which could have masked underlying physiological plasticity. In contrast to physiological traits, biomass and RGR were highly plastic, increasing in response to nutrient addition and decreasing in response to soil moisture. These responses indicate that plasticity, particularly in non-serpentine species, may help these species respond positively to nutrient deposition and negatively to increased precipitation under future environmental change scenarios.

### Effects of Phylogeny on Trait Responses

Although resource amendment was a stronger driver of trait responses than phylogeny, species within a congener pair responded more similarly to each other than species across congener pairs, especially in the *Quercus* species. The oaks, regardless of origin, responded very similarly to each other for all traits measured as observed in the PCA analysis, particularly along the first axis, suggesting that phylogeny influences how these species respond to resource availability. If the oaks share a more recent common ancestor than the other congener pairs, they would have had less time for trait divergence. However, dated phylogenies on the suite of species studied here are needed to investigate this hypothesis. Although each congener pair of *Arctostaphylos* and *Ceanothus* was similar in the direction of their trait responses, the magnitude of their responses between species within a pair differed. For example, the serpentine species of each pair had slower RGR and lower total biomass, irrespective of treatment, as is common among slow-growing species (Aerts and Chapin, 2000). These results are in agreement with other findings for traits exhibited by serpentine and nonserpentine species growing in the California chaparral, in which congener species behaved similarly in resorption proficiency (Drenovsky et al., 2013) and biomass and leaf nutrient concentrations (O’Dell et al., 2006). Furthermore, these data suggest that the oaks, an iconic genus of the California Floristic Province, may be less responsive to future environmental change than other taxa.

### Coordination Among Functional Traits

In general, larger, faster growing plants had greater nutrient pools and resorbed less N than plants from other treatments. However, due to low plasticity in morphological traits, faster growing species did not differ in root allocation or have higher specific leaf area, as would be predicted from the literature (Poorter and Remkes, 1990; James and Drenovsky, 2007). This result is surprising, as root investment is considered an important trait response supporting plant performance in arid and semi-arid environments (Funk et al., 2017). Instead, faster growth rates were associated with allocation of nutrient pools aboveground and a greater instantaneous phosphorus use efficiency. These responses stress the importance of mineral nutrient-related functional traits, which are often not included in trait-based analyses. Furthermore, these data suggest strong differences in nutrient sequestration capacity depending on soil water availability, with greatest storage capacity in these woody species associated with droughted soils.

## Conclusions

Climate change is one of the largest threats to biodiversity across the globe. Although it is not possible to directly use controlled environment studies to determine how species abundance and plant community composition may shift with climate change, our study provides new insight on how co-existing species may vary in sensitivity to changes in precipitation and nutrient availability by building off a well-developed plant functional trait framework. Namely, we show that in these nutrient-poor dryland systems nutrient allocation and nutrient use efficiency are key drivers of growth rate variation and plasticity. Overall, nonserpentine species generally had greater trait plasticity and thus may show greater biomass responses to anthropogenic-driven changes in resource availability, although there were well-defined evolutionary constraints on some of these responses. While we are not able to determine if these responses incur a fitness benefit or cost over the long term or how species-specific responses would scale to plant community or ecosystem level impacts, this study is a first step toward mechanistic insight on how co-existing species may vary in response to anthropogenic driven changes in soil resource availability. In these island-like serpentine systems, in which dispersal and migration is limited, these communities may be particularly susceptible to climate change factors and their constraints on recruitment and growth. Future work that combines long-term manipulations of resource availability and assessment of how key functional traits vary across plant communities are needed to identify how changes in soil resource availability may impact biodiversity, primary production, and nutrient storage within plant communities.

## Author Contributions

RN, RD conceived the idea with feedback from JJ. RN carried out the experimental work and statistical analyses. RN drafted the initial manuscript, which was jointly revised by RD, JJ.

### Conflict of Interest Statement

The authors declare that the research was conducted in the absence of any commercial or financial relationships that could be construed as a potential conflict of interest.

## Footnotes

^1^US Climate Data. (2017). Climate Lower Lake–California [Online]. Website: http://www.usclimatedata.com (Accessed: January 25, 2017).
