# Supplementary material for: Functional Traits Explain Variation in Chaparral Shrub Sensitivity to Altered Water and Nutrient Availability
Source: Front Plant Sci. 2019 Apr 18;10:505. doi: 10.3389/fpls.2019.00505 (PMC6482203; doi:10.3389/fpls.2019.00505)
Supplement: Supplementary file 1 [file Table_1.docx]

**Supplemental Table 1.** MANOVA results for physiological traits (A; photosynthetic rate, water use efficiency, photosynthetic N use efficiency, photosynthetic P use efficiency), biomass related traits (B; RGR, total biomass, root mass ratio, and specific leaf area), and tissue chemistry (C; green leaf N and P). For each trait group, the factors with the highest order significance are in bold. Non-significant model factors are indicated by “n.s.”

| **A. Physiological traits** |  |  |  |  |  |  |
| --- | --- | --- | --- | --- | --- | --- |
|  |  |  |  |  |  |  |
| **Model factor** | **df** | **Pillai's trace** | **dfn** | **dfd** | ***F*** | ***P*** |
| Water | 1 | 0.462 | 4 | 78 | 16.725 | <0.0001 |
| Nutrients | 1 | 0.449 | 4 | 78 | 15.905 | <0.0001 |
| **Origin** | **1** | **0.135** | **4** | **78** | **3.041** | **0.021** |
| **Species** | **4** | **1.359** | **16** | **324** | **10.425** | **<0.0001** |
| Block | 9 | 0.545 | 36 | 324 | 1.418 | n.s. |
| **Water*Nutrients** | **1** | **0.239** | **4** | **78** | **6.137** | **<0.0001** |
| Water*Origin | 1 | 0.061 | 4 | 78 | 1.270 | n.s. |
| Nutrients*Origin | 1 | 0.096 | 4 | 78 | 2.064 | n.s. |
| Water*Nutrients*Origin | 1 | 0.023 | 4 | 78 | 0.467 | n.s. |
|  |  |  |  |  |  |  |
| **B. Biomass and allocation traits** | |  |  |  |  |  |
|  |  |  |  |  |  |  |
| **Model factor** | **df** | **Pillai's trace** | **dfn** | **dfd** | ***F*** | ***P*** |
| Water | 1 | 0.41443 | 4 | 183 | 32.379 | <0.0001 |
| Nutrients | 1 | 0.40825 | 4 | 183 | 31.563 | <0.0001 |
| Origin | 1 | 0.39384 | 4 | 183 | 29.725 | <0.0001 |
| **Species** | **4** | **1.34582** | **16** | **744** | **23.578** | **<0.0001** |
| Block | 9 | 0.19035 | 36 | 744 | 1.033 | n.s. |
| **Water*Nutrients** | **1** | **0.17728** | **4** | **183** | **9.858** | **<0.0001** |
| **Water*Origin** | **1** | **0.06249** | **4** | **183** | **3.05** | **0.018** |
| **Nutrients*Origin** | **1** | **0.12822** | **4** | **183** | **6.729** | **<0.0001** |
| Water*Nutrients*Origin | 1 | 0.04298 | 4 | 183 | 2.054 | n.s. |
|  |  |  |  |  |  |  |
| **C. Leaf N and P** |  |  |  |  |  |  |
|  |  |  |  |  |  |  |
| **Model factor** | **df** | **Pillai's trace** | **dfn** | **dfd** | ***F*** | ***P*** |
| Water | 1 | 0.54738 | 2 | 156 | 94.328 | <0.0001 |
| Nutrients | 1 | 0.3989 | 2 | 156 | 51.763 | <0.0001 |
| Origin | 1 | 0.02694 | 2 | 156 | 2.16 | n.s. |
| **Species** | **4** | **0.74706** | **8** | **314** | **23.402** | **<0.0001** |
| Block | 9 | 0.14019 | 18 | 314 | 1.315 | n.s. |
| **Water*Nutrients** | 1 | 0.23685 | 2 | 156 | 24.208 | **<0.0001** |
| Water*Origin | 1 | 0.01422 | 2 | 156 | 1.125 | n.s. |
| **Nutrients*Origin** | **1** | **0.07577** | **2** | **156** | **6.394** | **0.002** |
| Water*Nutrients*Origin | 1 | 0.00457 | 2 | 156 | 0.358 | n.s. |
