# Supplementary material for: Functional Traits Explain Variation in Chaparral Shrub Sensitivity to Altered Water and Nutrient Availability
Source: Front Plant Sci. 2019 Apr 18;10:505. doi: 10.3389/fpls.2019.00505 (PMC6482203; doi:10.3389/fpls.2019.00505)
Supplement: Supplementary file 2 [file Table_2.DOCX]

**Supplemental Table 2.** ANOVA results for senesced leaf N (A), leaf lifespan (B), total N pool (C), total P pool (D), allocation of N to root pools (E), and allocation of P to root pools (F). Factors with the highest order significance are in bold. Non-significant model factors are indicated by “n.s.”

| **A. Senesced Leaf N** |  |  |  |
| --- | --- | --- | --- |
|  |  |  |  |
| **Model factor** | **df** | ***F*** | ***P*** |
| Water | 1 | 28.518 | <0.0001 |
| Nutrients | 1 | 9.27 | 0.003 |
| **Origin** | **1** | **5.338** | **0.023** |
| **Species** | **4** | **5.255** | **<0.0001** |
| Block | 1 | 0.238 | n.s. |
| **Water*Nutrients** | **1** | **4.236** | **0.043** |
| Water*Origin | 1 | 0.982 | n.s. |
| Nutrients*Origin | 1 | 1.742 | n.s. |
| Water*Nutrients*Origin | 1 | 2.103 | n.s. |
| Residuals | 79 |  |  |
|  |  |  |  |
| **B. Leaf lifespan** |  |  |  |
|  |  |  |  |
| **Model factor** | **df** | ***F*** | ***P*** |
| Water | 1 | 0.151 | n.s. |
| **Nutrients** | **1** | **6.948** | **0.0096** |
| Origin | 1 | 3.038 | n.s. |
| Species | 4 | 1.59 | n.s. |
| Block | 1 | 0.635 | n.s. |
| Water*Nutrients | 1 | 0.024 | n.s. |
| Water*Origin | 1 | 0 | n.s. |
| Nutrients*Origin | 1 | 1.173 | n.s. |
| Water*Nutrients*Origin | 1 | 0.675 | n.s. |
| Residuals | 115 |  |  |
|  |  |  |  |
| **C. Total N Pool** |  |  |  |
|  |  |  |  |
| **Model factor** | **df** | ***F*** | ***P*** |
| Water | 1 | 129.869 | <0.0001 |
| Nutrients | 1 | 113.955 | <0.0001 |
| Origin | 1 | 25.235 | <0.0001 |
| **Species** | **4** | **40.061** | **<0.0001** |
| Block | 1 | 3.095 | n.s. |
| **Water*Nutrients** | **1** | **22.293** | **<0.0001** |
| **Water*Origin** | **1** | **7.286** | **<0.0001** |
| **Nutrients*Origin** | **1** | **7.493** | **0.0069** |
| Water*Nutrients*Origin | 1 | 0.01 | n.s. |
| Residuals | 155 |  |  |
|  |  |  |  |
| **D. Total P Pool** |  |  |  |
|  |  |  |  |
| **Model factor** | **df** | ***F*** | ***P*** |
| Water | 1 | 36.214 | <0.0001 |
| Nutrients | 1 | 18.467 | <0.0001 |
| Origin | 1 | 5.341 | 0.0228 |
| **Species** | **3** | **7.686** | **0.0001** |
| Block | 1 | 3.457 | n.s. |
| Water*Nutrients | 1 | 0.614 | n.s. |
| Water*Origin | 1 | 0.037 | n.s. |
| **Nutrients*Origin** | **1** | **4.435** | **0.0376** |
| Water*Nutrients*Origin | 1 | 0.462 | n.s. |
|  | 106 |  |  |
|  |  |  |  |
| **E. Allocation of N to Root Pools** | |  |  |
|  |  |  |  |
| **Model factor** | **df** | ***F*** | ***P*** |
| **Water** | **1** | **27.615** | **<0.0001** |
| **Nutrients** | **1** | **32.138** | **<0.0001** |
| Origin | 1 | 2.943 | n.s. |
| **Species** | **4** | **45.725** | **<0.0001** |
| Block | 1 | 0.006 | n.s. |
| Water*Nutrients | 1 | 0.034 | n.s. |
| Water*Origin | 1 | 0.078 | n.s. |
| Nutrients*Origin | 1 | 1.753 | n.s. |
| Water*Nutrients*Origin | 1 | 0.843 | n.s. |
| Residuals | 155 |  |  |
|  |  |  |  |
| **F. Allocation of P to Root Pools** | |  |  |
|  |  |  |  |
| **Model factor** | **df** | ***F*** | ***P*** |
| Water | 1 | 36.166 | <0.0001 |
| Nutrients | 1 | 30.554 | <0.0001 |
| Origin | 1 | 17.82 | <0.0001 |
| **Species** | **3** | **101.029** | **<0.0001** |
| Block | 1 | 9.948 | 0.002 |
| Water*Nutrients | 1 | 1.407 | n.s. |
| **Water*Origin** | **1** | **7.56** | **0.007** |
| **Nutrients*Origin** | **1** | **5.003** | **0.027** |
| Water*Nutrients*Origin | 1 | 0.223 | n.s. |
| Residuals | 106 |  |  |
